# Nutrition of very low birth-weight infants

**DOI:** 10.1186/1824-7288-40-S1-A39

**Published:** 2014-08-11

**Authors:** Mario De Curtis, Lucia Dito, Renato Lucchini, Gianluca Terrin

**Affiliations:** 1 Department of Pediatrics and Neuropsychiatry, University of Rome La Sapienza, Rome, Italy; 2 Department of Gynecology-Obstetrics and Perinatal Medicine, University of Rome La Sapienza, Rome, Italy

## Background

Numerous studies have underlined the importance of early feeding on short- and long-term development of very low birth-weight (VLBW) neonates [1,2]. Nutrition of preterm infants may be divided in two subsequent periods: the early adaptive or “transition” period from birth to the second week of life followed by the “stable-growing” period up to discharge from the neonatal unit. Depending on birth weight and gestational age the transition period may be prolonged. Immediately after birth most VLBW infants are unable to start enteral feeding. Parenteral nutrition is presently proposed since the first days of life to limit malnutrition. The ideal of nutrients intake for VLBW neonates is still a matter of debate [1,2].

## Materials and methods

We revised studies with high level of evidence (randomized clinical trials and meta-analysis) regarding enteral and parenteral nutrition in VLBW neonates. Database: MEDLINE and Pubmed. Search term: Nutrition (AND) Very Low Birth Weight. Limits: Randomized clinical trial (AND) Meta-analysis.

## Results

We analyzed 66 manuscript (12 meta-analysis, 44 RCT) published from 1984 to 2014. Analysis of the best evidences revealed that, up to now, early nutritional strategies vary dramatically among centers and there are no definitive and well accepted regimens demonstrating long term benefits in VLBW infants. To reduce the temporary interruption of the transfer of nutrients, a so called “aggressive” nutrition has been proposed, consisting in a high protein supply (>2g amino acids/kg/d) and the use of IV lipid (0.5-1.0 g lipid/kg/d), from the first day of life. Minimal enteral nutrition will be added as early as possible to provide a small quantity of milk to stimulate the enterocytes [3]. As the infant matures and the medical conditions stabilize parenteral nutrition can be slowly replaced by enteral nutrition. Nutrition of VLBW infants during the transition period should cover as much as possible the nutritional needs, to limit the inevitable cumulative nutritional deficit, induce a positive nitrogen balance and reinitiate weight gain and longitudinal growth. During “stable-growth” in most preterm infants nutritional supplies can be provided by fortified mother’s milk or preterm formulas.

## Conclusions

Analysis of best evidences suggest that, in order to specifically enhance lean mass growth and improve the early catch up growth, the use of a high protein regimen (4.0-4.4 g of protein/kg/d) combined with a high protein energy density regimen (protein/energy ratio up to 3.3 g/100 kcal) early in life promotes growth without metabolic stress in VLBW infants.
